# Improving the planning of the GP workforce in Australia: a simulation model incorporating work transitions, health need and service usage

**DOI:** 10.1186/s12960-016-0110-2

**Published:** 2016-04-11

**Authors:** Caroline O. Laurence, Jonathan Karnon

**Affiliations:** School of Public Health, The University of Adelaide, North Terrace, Adelaide, Australia

**Keywords:** General practice, Health workforce, Health needs, Utilisation, Simulation model

## Abstract

**Background:**

In Australia, the approach to health workforce planning has been supply-led and resource-driven rather than need-based. The result has been cycles of shortages and oversupply. These approaches have tended to use age and sex projections as a measure of need or demand for health care. Less attention has been given to more complex aspects of the population, such as the increasing proportion of the ageing population and increasing levels of chronic diseases or changes in the mix of health care providers or their productivity levels. These are difficult measures to get right and so are often avoided. This study aims to develop a simulation model for planning the general practice workforce in South Australia that incorporates work transitions, health need and service usage.

**Methods:**

A simulation model was developed with two sub-models—a supply sub-model and a need sub-model. The supply sub-model comprised three components—training, supply and productivity—and the need sub-model described population size, health needs, service utilisation rates and productivity. A state transition cohort model is used to estimate the future supply of GPs, accounting for entries and exits from the workforce and changes in location and work status. In estimating the required number of GPs, the model used incidence and prevalence data, combined with age, gender and condition-specific utilisation rates. The model was run under alternative assumptions reflecting potential changes in need and utilisation rates over time.

**Results:**

The supply sub-model estimated the number of full-time equivalent (FTE) GP stock in SA for the period 2004–2011 and was similar to the observed data, although it had a tendency to overestimate the GP stock. The three scenarios presented for the demand sub-model resulted in different outcomes for the estimated required number of GPs. For scenario one, where utilisation rates in 2003 were assumed optimal, the model predicted fewer FTE GPs were required than was observed. In scenario 2, where utilisation rates in 2013 were assumed optimal, the model matched observed data, and in scenario 3, which assumed increasing age- and gender-specific needs over time, the model predicted more FTE GPs were required than was observed.

**Conclusions:**

This study provides a robust methodology for determining supply and demand for one professional group at a state level. The supply sub-model was fitted to accurately represent workforce behaviours. In terms of demand, the scenario analysis showed variation in the estimations under different assumptions that demonstrates the value of monitoring population-based need over time. In the meantime, expert opinion might identify the most relevant scenario to be used in projecting workforce requirements.

**Electronic supplementary material:**

The online version of this article (doi:10.1186/s12960-016-0110-2) contains supplementary material, which is available to authorized users.

## Background

The aim of health workforce planning is to have the right skills in the right place at the right time to provide the right services to the right people [1, 2]. This process involves the estimation of future requirements for health care and the supply of resources to provide the required services and consideration and testing of policy options to address any predicted differences between demand and supply. Three concepts may be used to inform the required services. ‘Utilisation’ describes the use of available services, ‘demand’ describes service use if access could be optimised and ‘need’ defines service use if all health care would be accessed by patients for whom treatment is appropriate.

It is difficult to predict the future of the health care system, and so, applied approaches are based on varying assumptions that reflect the modelling methodology and the available data. When these planning assumptions are incorrect they can create problems. Currently, there are insufficient pre-vocational clinical training positions in South Australia for the increased number of new medical graduates arising from an expansion of medical school places, which requires expensive strategies to resolve [3]. We have seen the results of poor planning methods in Australia with the swings from perceived oversupply of general practitioners (GPs) in the 1980s to undersupply in the 1990s, the reliance on overseas-trained doctors and large increases in locally trained medical graduates from 2012 onwards [4].

In Australia, as in many developed countries, workforce planning has been based on workforce benchmarks [5, 6], such as GP to population ratios which are then applied to predicted changes in the size and age of the future population. The most recent health workforce planning work undertaken by Health Workforce Australia still has this focus [7–9]. These approaches have incorporated some demographic projections, primarily simply by age and sex, with the assumption that current utilisation rates are appropriate and changes in population size and structure determine changes in required services [1, 8, 9].

These existing models facilitate the consideration of supply-side issues, such as training, recruitment, retention, career paths and practice activity but provide less opportunity for considering other policy options, in particular options for representing the need and influencing the demand for health services [10]. Little attention has been given to the more complex aspects of population structure, such as the increasing proportion of the population with chronic diseases, to changes in the productivity of providers, or provider incentives in a fee-for-service system, and their responses to under- or oversupply. These are difficult components to incorporate in a planning model and so are often avoided, but there are methods that can be applied to this task.

### A need-based approach to planning the health workforce

One option is to take a need-based approach which estimates future requirements based on estimated health deficits of the population [11]. It determines age- and sex-specific service needs based on service norms and morbidity trends. In Canada, Birch and Tomblin Murphy [2, 12–18] developed and applied a need-based approach to health workforce planning. The conceptual framework they developed for the model acknowledges the complexity of workforce planning in health, in an environment that is influenced by economic, social, political, technological and geographical factors.

### Determining need

A key component of the need-based approach is to define and measure ‘need’. Tomblin Murphy et al. identified four categories of need constructs—measures of health risk, measures of morbidity, measures of mortality and measures of subjective health status [19]. Self-reported health status is known to correlate with a range of health outcomes and socio-economic variables [20–23] and has been shown to be a predictor of mortality and visits to the doctor or hospital [16]. Tomblin Murphy and colleagues [18, 24, 25] used self-reported health status as a measure of need when estimating the required number of health professionals including primary care professionals. They argued that it is an individual’s own assessment of their health status that leads to initial consultations with primary care providers.

Mortality, morbidity, prevalence and incidence rates are used in epidemiology to provide information on the burden of disease within the population as well as risk factors for future illness. Prevalence and incidence rates have been used to determine the need for mental health services in Australia [26] and physician supply in Canada [27], with the latter also incorporating risk factors. In Australia, the Northern Territory government used a need-based approach in planning hospital services using disability-adjusted life years as a measure of need [28].

Most of these models have focused on the tertiary setting, and very few apply the models to the primary care setting. This arises mainly from the lack of data required to populate the model and the complexity of modelling primary care needs. The aim of this study was to develop a simulation model for planning the GP workforce in South Australia (SA) that incorporates population health needs with estimated demand. The developed model is intended to estimate the future supply of GPs, as well as the need for GP services in SA, and to facilitate scenario analyses to test the impact of varying assumptions regarding changes to supply and need in the future.

## Methods

### Model overview

**Fig. 1:**
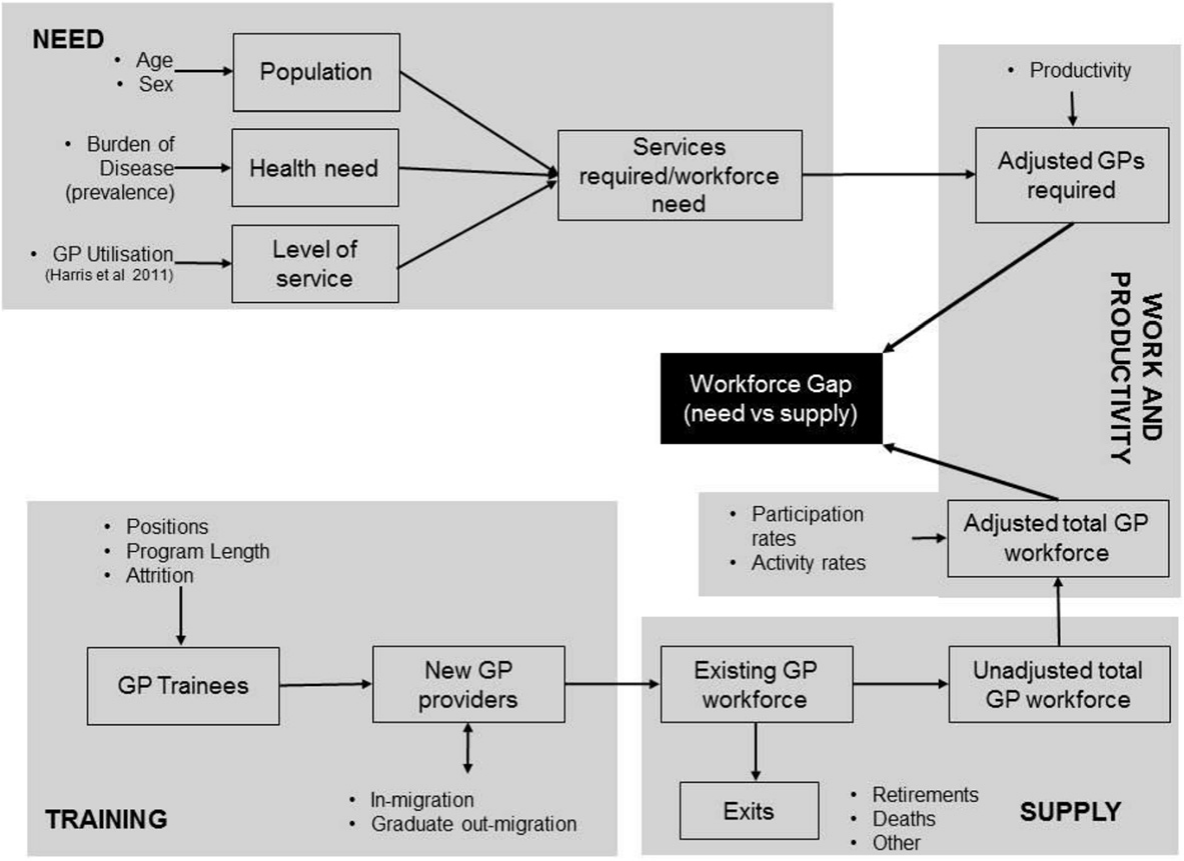
Overview of the planning model for GP

A deterministic workforce planning simulation model was developed, comprising two sub-models which describe the supply and demand for GPs, incorporating components of the four modules used by Tomblin Murphy et al. [18] (see Fig. 1).

The supply sub-model describes the movement of the existing GP stock between part-time and full-time status, and rural and urban locations, as well as the entry and exit of GPs from the existing stock. The demand model combines disease incidence and prevalence data with age- and gender-specific utilisation rates per incident/prevalent case to estimate the aggregate level of services required over the whole population and the number of GPs required to provide the required level of services. The baseline values used for the key parameters are shown in Table 1.

**Table 1:** Baseline values used for the simulation model for GPs in South Australia

Module	Parameter	Baseline value (2003)
Training	Graduates	42
Supply	In-migration	47 (from interstate or overseas)
	Existing provider stock	1 789 (headcount) 1 603 (FTE)
	Exit rates (temporary and permanent)	See Additional file 1 for rates by age, sex and location
Work and productivity	Productivity	1 760 consulting hours per FTE GP (based on 40 h per week)
Needs	Population	1 529 424
	Need	Prevalence and incidence cases for a range of health conditions
	Level of service	Number of consultations per person per year by age, sex and health condition

The base years for the supply and need sub-models were 2004 and 2003, respectively, and input parameters for both sub-models were fitted to match observed data beyond the base years.

### Data sources

The data to populate the model was sourced from a range of organisations. The scope of these data sources varied considerably in terms of the time period covered, the available units of measurement and aspects of the workforce considered. Where necessary, customised data were provided on request or purchased to meet the specific requirements of the model. The main datasets, their source and variables used for this model are outlined in Table 2. The data were then analysed to identify key trends occurring within the population and SA GP workforce. This included an analysis of changes within the GP registrar profiles and completion rates, changes in hours worked by GPs and changes in the number of GP consultations. A reference group, comprising representatives and experts from key organisations, also provided additional information on trends within the GP workforce.

**Table 2:** Main datasets and variables used in the simulation model for the GP workforce

Module	Dataset	Organisation	Description and scope of dataset	Variables used
Training	Australian General Practice Training (AGPT) programme	General Practice Education and Training (GPET) Ltd.	Up until December 2015, GPET managed the Australian General Practice Training programme which is the largest GP training programme in Australia with 1 200 positions available nationally in 2014 and increasing to 1 500 in 2015. The programme is funded by the Australian Government, and places are allocated to regional training providers across the country. Selection and subsequent allocation of registrars is undertaken nationally. GPET maintained data on all registrars enrolled in the training programme from 2002 to 2015.	Training places and enrolments by state; Graduate numbers by age^a^ and sex
Supply	Medical Labour Force Surveys (custom data)	Australian Institute of Health and Welfare (AIHW)	The AIHW has undertaken Medical Labour Force Surveys in Australia since 1997. These surveys collect information on the demographic and employment characteristics of all medical practitioners in Australia who were registered at the time of the survey.	Number of GPs—age^a^, sex, location (rural or urban) and work status (full-time or part-time)
	GP workforce statistics	Australian Government Department of Health (Medicare)	Claims data collected by Medicare Australia is also used to report on the Australian GP workforce, and these are available annually. Data is provided on the number of GPs by state, age, sex and work status.	Number of GPs—age^a^, sex, location (rural or urban), type and work status (full-time or part-time)
	International medical graduates	Rural Doctors Workforce Agency (RDWA)	The RDWA is based in South Australia and is one of seven rural workforce agencies in Australia funded by the Department of Health. The core activities of the agency are recruitment, support services and workforce planning for rural and remote communities. The RDWA manages the recruitment of GPs to rural and remote communities, and they undertake a survey of all rural and remote GPs in SA every 3 years. This survey includes information on the characteristics of the GPs, working hours and retention issues.	Number of IMGs—age^a^, sex, work status (full-time or part-time)
Work and productivity	Medicare Australia and Department of Veteran Affairs	Family Medicine Research Centre, University of Sydney	A random sample of approximately 1 000 GPs participating in the programme in 2006.	GP utilisation rates—age^a^ and sex
Need	Burden of Disease study	Australian Institute of Health and Welfare	The Burden of Disease and Injury in Australia study [32] published in 2007 was a comprehensive assessment of the health status of Australians. The study measured mortality, disability, impairment, illness and injury arising from 176 diseases, injuries and risk factors using a common metric, the disability-adjusted life year or DALY, and methods developed by the Global Burden of Disease Study. Annexes to the report provide data on a number of measurements including incidence and prevalence, and these were used for this study.	Prevalence cases and incidence cases by age^a^ and sex
	SA population projections	Australian Bureau of Statistics	The ABS is Australia’s national statistical agency. It provides statistics on a number of key indicators such as housing, economy, environment and energy. It also manages and analyses the Australian Census of Population and Housing every 5 years. It also provides population projections at a national and regional level.	Age^a^ and sex
	Bettering the Evaluation and Care of Health (BEACH)—GP activity (customised data)	Family Medicine Research Centre, University of Sydney	The BEACH Program is a continuous national study of GP activity in Australia which commenced in 1998. Each year, a random sample of approximately 1 000 GPs participates in the programme. They record information on 100 consecutive patient encounters [46]. The source population for sampling includes all vocationally registered GPs and all general practice registrars in Australia who claimed a minimum of 375 Medicare general practice items of service in the most recently available 3-month Medicare data period. The data used was a weighted sub-sample of SA GPs in 2003.	Problems managed by SA GPs by age^a^ and sex
	GP attendances	Medicare Australia	Medicare Australia data includes services that qualify for a Medicare Benefit under the Health Insurance Act 1973 and for which a claim has been processed by the Department of Human Services. Its cover includes data on services provided by all active Australian medical practitioners eligible for claiming medical benefits.	Unreferred attendances by Broad Type of Services for SA by age^a^ and sex

^a^Age groups: <35 years, 35–44 years, 45–54 years, 55–64 years, 65+ years

#### GP stock

Data on the SA GP stock were obtained from the 2004 Australian Institute of Health and Welfare (AIHW) medical workforce survey. Workforce status was derived from the AIHW categories of hours worked for each age group and sex. Part-time was defined as less than 35 h and full-time as equal to 35 h or more per week. Location was defined as either urban or rural, based on the Australian Standard Geographical Classification Remoteness Areas [29]. This classification system has five categories (RA1 to RA5), and for this study, they were collapsed into two: RA1 (major cities), defined as urban, and RA2–RA5 (inner regional, outer regional, remote and very remote), defined as rural. This approach is commonly used for a broad definition of urban and rural areas in Australia and offset confidentiality issues for data for remote and very remote areas.

#### Temporary and permanent exit rates

Temporary and permanent exit numbers for the SA GP stock in the base year were not able to be determined from the AIHW labour force data. The Medicine in Australia: Balancing Employment and Life (MABEL) longitudinal dataset [30] was used as a reference data source to inform the probabilities of temporary and permanent exits from the workforce in the base year.

#### Population estimates

Observed and projected population estimates for the SA population by age and sex were obtained from the Australian Bureau of Statistics (ABS) [31]. The ABS provides three population projections based on different assumptions on net population changes (e.g. fertility, migration and life expectancy) and are termed series A, B and C. Series A assumes higher levels of components of population change while series C assumes lower levels. As a midpoint, series B was used in the model.

#### Incidence and prevalence data

Data on incidence and prevalence across disease and injury groups were available at a national level through the 2003 Australian Burden of Disease study [32]. These data are coded into categories based on the International Classification of Disease Version 10 (ICD-10) and included diseases and injuries grouped according to cause under three broad categories: communicable disease, maternal and neonatal conditions (e.g. acute respiratory infections, infectious diseases); non-communicable diseases (e.g. diabetes mellitus, malignant neoplasms, mental disorders); and injuries (e.g. unintentional and intentional injuries). A full list of the health conditions included in the incidence and prevalence data are available from the authors. The national data were used as the prevalence and incidence data used in the SA Burden of Disease study were less comprehensive.

#### Levels of service requirements

There is currently no ‘gold standard’ on the level of services/consultations required for individuals with different levels of need or health conditions. Therefore, we fitted age- and sex-specific numbers of consultations per person with alternative health conditions for the base year (2003), using data describing the total numbers of consultations, and the problems managed within those consultations.

The average number of consultations per year for the SA population in each age and sex group was based on Medicare Australia claims data using unreferred attendances for GP items grouped by Broad Type of Service (BTOS). Unreferred attendances are Medicare items that relate to GP services and represent consultations. For this analysis, attendances were based on three BTOS categories—vocationally registered GP, other GP and enhanced primary care.

The Bettering Evaluation and Care of Health (BEACH) study provided data describing the proportion of consultations in SA in 2003 in which alternative health conditions were managed. Seventeen managed health conditions were classified according to the International Classification of Primary Care version 2 (ICPC-2) chapter headings [33]. In addition to the management of existing problems, a proportion of consultations involve preventative and administrative activities. To account for this activity, we estimated the proportion of services (by age and sex) involving ICPC-2 codes relating to such activities (e.g. immunisations, health checks and administrative procedures).

To combine the need and utilisation data, the incidence and prevalence data were recoded into the ICPC-2 codes. Within each age and sex group, the proportions of consultations involving each of the 17 health conditions were divided by the numbers of incident and prevalent cases of the condition to estimate the average annual consultations per case. The average numbers of consultations involving preventative and administrative activities were estimated across the whole of the population for each age and sex group.

The average length of consultation for each age and sex group was based on research undertaken by Harrison and Britt [34]. When converting the estimated number of consultations per year to the required number of full-time equivalent (FTE) GPs, a standard working week of 40 h of clinical work was used. This is used by AIHW when analysing the medical workforce surveys [35]. A 44-week working year was selected to allow for various types of leave such as sick leave, training, annual leave and public holidays.

### Supply sub-model

The supply sub-model used a stock and flow approach to estimate the future GP headcount based on existing numbers of GPs working in clinical care. The supply of GPs was based on the stock of potential GPs who are qualified to provide services and the flow of provider time from the stock, i.e. their participation and activity rates.

In estimating the future size of the GP stock, the following factors were included:

- The current stock of GPs which included all GPs who were available to provide care (active in clinical practice)
- Entry to the stock of new graduates of GP vocational training programmes and entrants from other locations including migration
- Exit rates from the stock which included permanent exits (e.g. deaths, retirements) and temporary exits (e.g. maternity leave).

The GP stock was estimated by age, sex, location (rural/urban) and work participation levels, i.e. part-time and full-time status of working GPs. These variables were selected because of their relevance to the Australian GP workforce. In Australia, geographic maldistribution is a major workforce issue, with insufficient numbers of GPs working in rural and remote areas and, as a result, the focus of many government policies [36]. We also know that the participation rates of GPs are changing, with female GPs working less hours than male GPs and male GPs working less over a period of time, and that these rates vary across age groups [37]. As a result, these variables are important considerations in GP workforce planning.

#### Base year GP stock component

The headcount of the base year GP stock was converted to FTE by multiplying the headcount by part-time or full-time hours per week divided by a standard full-time clinical working week. This method was used for calculating FTE for all other years in the model (2004–2013).

#### Future GP stock component

**Fig. 2:**
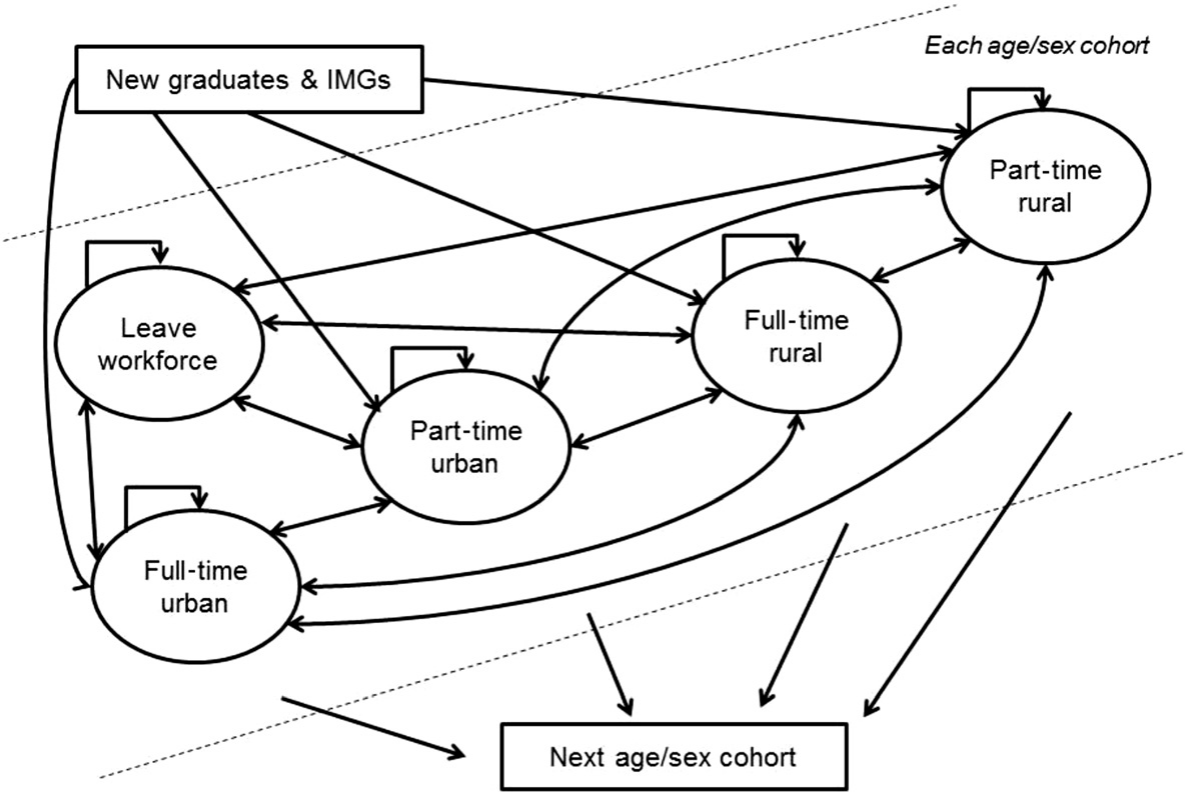
Summary of the state and transition model for the GP supply sub-model for each cohort

To estimate the future GP stock in SA, the approach taken was to build a cohort-based state transition model [38]. The states in the model were the following: male and female GPs by age groups (<35 years, 35–44 years, 45–54 years, 55–64 years, 65+ years), locality (rural and urban), workforce status (full- or part-time) and exits (permanent and temporary). The complete set of model states used in the sub-model are outlined in Fig. 2, as well as the possible transitions that could be made between states. The model works by estimating the proportion of GPs in each state at the beginning and end of each calendar year. During each year, transition probabilities were applied that determine the movement of GPs between the states. For example, if there are 100 male GPs working full-time in urban areas at the beginning of a year, and the annual transition probability for full-time urban GPs to part-time urban GPs is 0.05, then five GPs (0.05 × 100) will move from the full-time urban GP state to the part-time urban GP state in that year.

#### Estimating the transition probabilities

Annual transition probabilities, by age and sex, reflected mobility (movement between urban and rural practice), participation (movement between full-time and part-time) and exits from the workforce (temporary and permanent), as described in Additional file 1. Beta (Bayesian prior) probability distributions were used to represent the uncertainty around each of the transition probabilities in the supply sub-model, initially based on data provided by the MABEL longitudinal study [30]. Data were extracted from five waves (years) for GPs in each employment state (e.g. full-time urban males, aged 35–44 years). From this, we determined the proportion of GPs who remained in the same state and who moved to another state, e.g. moved from full-time rural to part-time rural.

A set of transition probabilities for the supply sub-model was sampled from the prior probability distributions, which were applied to the GP stock in the base year (2003). To allow for variation in transition probabilities over time, multiplier parameters were sampled for each transition probability, which described an annual percentage change in each transition probability. The multipliers were sampled from uniform probability distributions ranging from −10% to +10%.

#### Calibration of supply sub-model

Sets of transition probabilities for the supply sub-model were selected from the prior probability distributions that best predicted the observed GP stock data. Calibration targets for the model comprised estimates of GP numbers, by age, gender, work status (full-time and part-time) and location, as reported by the AIHW medical workforce surveys for the period 2004–2011 (2011 was the latest year of available data at the time of the study). Over the period, 2004–2011, there were changes in the definitions used to define hours worked and part-time and full-time status, and so, the data were smoothed across the time horizon by estimating the trends for each GP stock output parameter, e.g. full-time, urban, male GPs aged under 35 years. Pre-specified convergence criteria required differences of less than 5% between predicted and observed values across all calibration targets.

The model was built in Excel, using the add-in Premium Solver Pro 2014 to implement a search algorithm to identify sets of convergent input parameter values from the prior probability distributions.

The model did not fit the observed GP stock data well using the MABEL-based prior probability distributions. An analysis of the transition probabilities using the MABEL data-resultant supply sub-model outcomes were presented to the reference group, and it was decided that the MABEL data were thought to overestimate the probability of transition between employment states, partly a result of the bias in the respondent population. Therefore, we reviewed the transition probabilities manually to adjust the specified ranges for each transition probability, which resulted in a fitted model that met the pre-specified convergence criteria.

Multiple sets of convergent input parameter values were identified, representing the joint uncertainty around the input parameters, which will inform sensitivity analyses around the planned scenario analyses of the forecasted supply and demand of GPs.

### Need sub-model

The need sub-model’s four components were population, health need, level of service and productivity. Three scenario analyses were specified to test alternative assumptions reflecting potential changes in need over time and appropriate utilisation rates.

In scenario one, we made the assumption that utilisation rates in 2003 reflected optimal use for the estimated burden of disease and that age- and gender-specific incidence and prevalence rates remained constant over time, i.e. changes in service requirements were driven solely by changes in population demographics.

In scenario two, we also assumed that age- and gender-specific incidence and prevalence rates remained constant over time but that optimal utilisation rates were achieved in 2013. To match utilisation and need in 2013, an annual multiplier of 1.12% for service use per incident/prevalent case was applied from 2003 to fit model predictions to the observed number of consultations between 2003 and 2013.

In scenario three, utilisation rates were assumed to be optimal in 2003, but age- and gender-specific incidence and prevalence rates increased over the modelled 10-year period. We applied an annual increase of 2% incidence and prevalence rates.

**Fig. 3:**
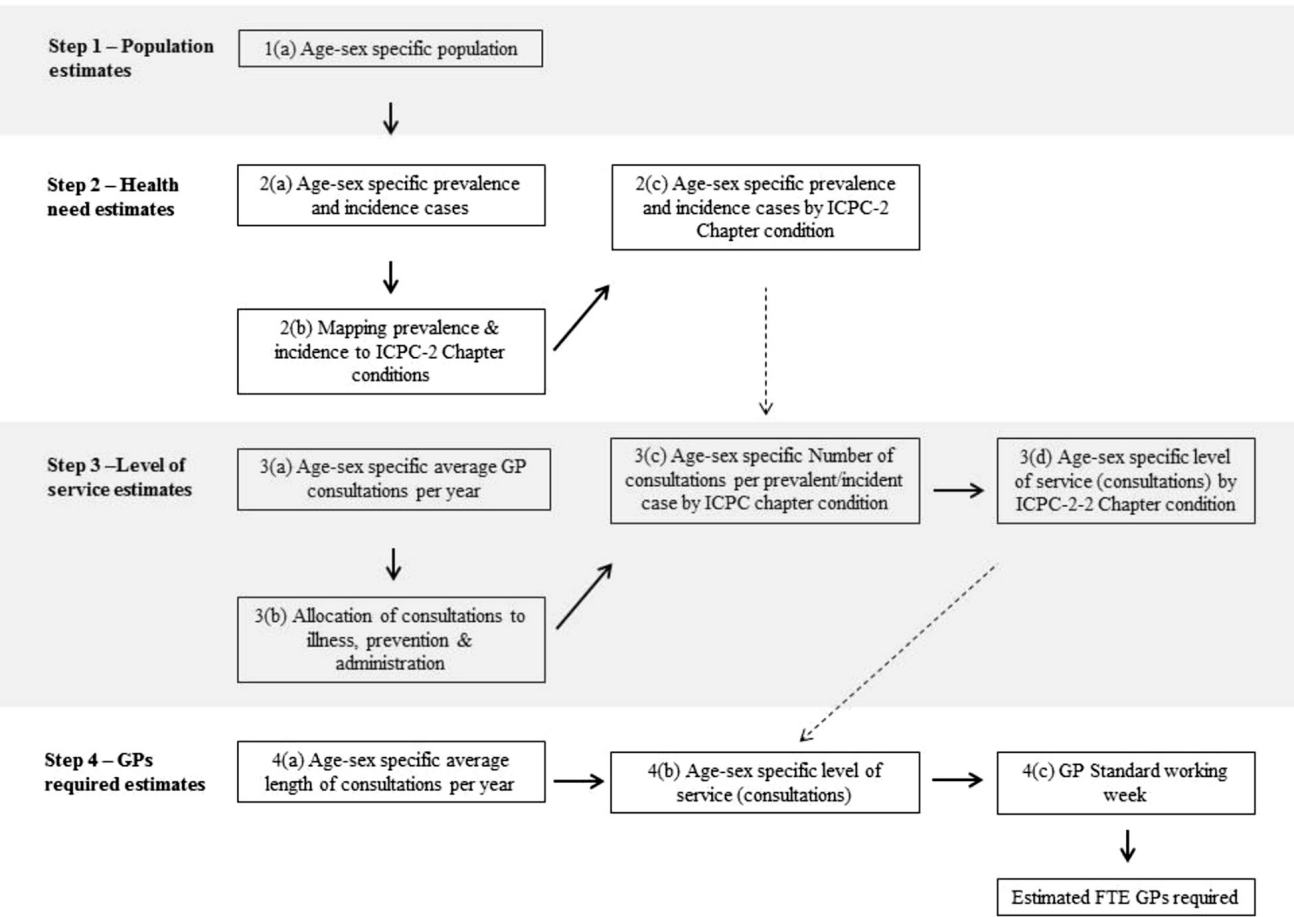
Summary of key steps in determining the estimates for the need sub-model

The key steps involved in the estimation of the need sub-model are shown in Fig. 3 and explained in detail below.

Annual estimates of the size and age/sex distribution of the SA population were informed by the Australian Bureau of Statistics. Disease incidence and prevalence in 2003 was informed by a major burden of disease study [32]. Annual numbers of consultations were estimated per prevalent/incident case for each of the 17 ICPC-2 categories, and the number of preventative and administrative consultations was estimated by age and sex. The number of consultations required for each ICPC-2 chapter heading by age and sex group was estimated for subsequent years by multiplying the annual number of consultations per prevalent/incident case by the prevalence/incidence estimates for each year (Fig. 3). The number of preventative/administrative consultations increased in line with population changes.

The final component of the need sub-model was to determine the number of FTE GPs required to provide the services to meet the health needs of the population. The average length of consultation (minutes) for each age and sex group was multiplied by the corresponding, estimated numbers of consultations required. Based on this, the required numbers of FTE GPs in SA were estimated for each year of the model (Fig. 3).

The Medicare Australia data on unreferred attendances for GP items grouped by BTOS for the period 2003–2013 informed the observed number of consultations in this period.

## Results

**Fig. 4:**
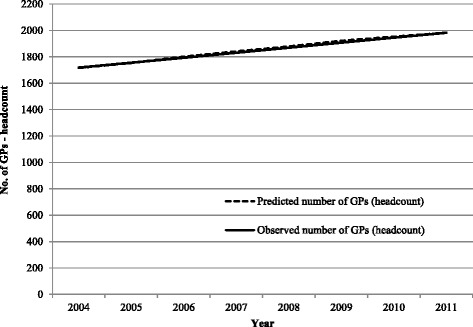
Comparison of predicted number of GPs (headcount) with observed (AIHW) number of GPs (headcount), South Australia, 2004–2011—supply sub-model

The comparison of actual versus predicted numbers of FTE GPs and number of GP consultations for the supply sub-model is shown in Fig. 4. For the supply sub-model, the general shape of the curve for the predicted number of GPs is similar to the observed data over the period 2004–2011 (Fig. 4). The model tends to overestimate the number of GPs required over this time horizon but the difference is small. In the base year 2004, the best-fitting supply sub-model estimated 1717 required GPs while the actual number of GPs was 1719, a difference of two general practitioners. However, by the end of the projection period (2011), this gap had decreased with the sub-model predicting the same number of GPs as was observed (1983 GPs). Over the 8-year period, the sum of the absolute differences between the observed and predicted supply of GPs was 49, a difference of 4.22% and within the set goal of 5% limit for a small difference.

**Fig. 5:**
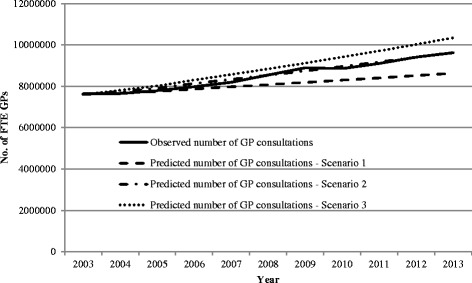
Comparison of estimated number of FTE GPs required with observed number of FTE GPs (Department of Health), South Australia, 2003–2013—for three scenarios: need sub-model

**Fig. 6:**
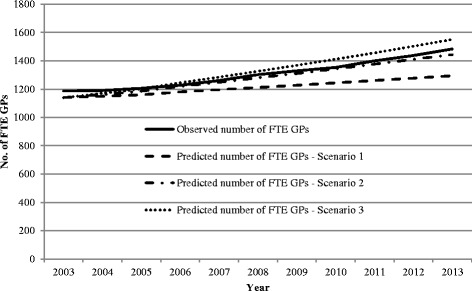
Comparison of estimated number of GP consultations with observed number of GP attendances (Medicare Australia), South Australia, 2003–2013—for three scenarios: need sub-model

The comparison of the actual versus predicted number of FTE GPs and GP consultations for the three need sub-model scenarios are shown in Figs. 5 and 6.

For all the scenarios, the model estimated at the required number of FTE GPs in the base year was 1140, while the actual number of FTE GPs was 1187, a difference of 47 FTE GPs (Fig. 5). Similarly, the model estimated that the required number of GP consultations in the base year was 7 607 928, while the actual number of consultations was 7 647 560, a difference of 39 632 consultations (Fig. 5). However, over the projection period, these estimations varied with each scenario.

In the first scenario, the difference between the observed and predicted FTE GPs increased with the model estimating 1294 FTE GPs required in 2013, 189 fewer GPs than reported by the Department of Health GP Workforce statistics from Medicare Australia [39].

For scenario 2 where the model assumed optimal utilisation rates were achieved in 2013, the difference between the estimated number of FTE GPs required and the actual number of GPs was small over the projection period. The difference between observed and predicted in 2013 was 39 FTE.

In the final scenario where there was an annual increase in the incident and prevalent cases over the projection period, we found that the model estimated a greater number of FTE GPs were required than occurred (1551 FTE GPs versus 1483 FTE GPs), a difference of 68 GPs.

The trends observed in GP numbers required in the model directly reflected the model estimations for the level of services required, with scenario 1 estimating fewer consultations than observed and scenario 2 estimating a similar number to that observed, and in scenario 3, the model estimated an increased number of consultations than observed (Fig. 6).

## Discussion

The main objective of this study was to develop a novel modelling approach to planning the GP workforce in Australia, which accounts for both demand and supply factors. This paper describes the estimation of the model’s input parameters and the comparison of the model’s outputs to 10 years of observed data between 2003 and 2013. Subsequent model analyses will predict changes in demand and supply into the future, to explore the effects of alternative population scenarios and policy options on projections of demand and supply.

In terms of the future supply of GPs, a state transition cohort model was used to predict the entry, exit and movement of GP stock. The application of a state transition model methodology in the supply sub-model allowed for a number of career transitions, which represents the behaviour of the GP workforce and provides more sophisticated scenario simulations. This approach accommodates some of the unique aspects of the GP stock—the increasing proportion of females entering this discipline and the associated increase in part-time work patterns. In Australia, GP training has the greatest proportion of female trainees of all medical specialities (64% in 2013) [40], and we know that it is likely that a large proportion of these trainees will work part-time for the majority of their career. Therefore, any simulation model designed to estimate the future GP supply should use a method that captures the changing work patterns of GPs.

Previous GP workforce planning models in Australia have not reflected population health needs. The GP to population ratios used in the 1990s [5] identified geographic maldistribution of the workforce, but these models could not explore the interactions between different parameters such as productivity, skill mix and social change, making it difficult to test different policy levers. The work undertaken by HWA produced a more complex workforce planning model which used a utilisation method to develop workforce demand projections [7, 8]. Expressed demand growth rates were developed for each medical speciality and scenario analysis undertaken to assess the impact on workforce projections of different policy options. Their model did not explicitly incorporate health need, but utilisation rates were adjusted for projected changes in the size and structure of the population. In the several health workforce simulation models developed recently in the USA, the models have used a utilisation approach in measuring demand for service but incorporated chronic conditions, smoking status and body weight into determining number of visits [41–43]. The model described here is similar to the US approach and provides a further advancement on the previous methods used in Australia by basing estimates of future health requirements on the basis of projected health and service needs of the population. Similarly, the Health Resources and Services Administration calibrated their model with other data sources in order to assess its external and predictive validity [43].

The results from the supply sub-model provided a reasonable fit to the observed data for the stock of GPs, while for the need sub-model, the difference between the observed and predicted number of GPs and consultations varied depending on the assumptions made.

The need sub-model estimated average age-, gender- and condition-specific utilisation rates, combined with age- and gender-specific incidence/prevalence data to predict aggregate utilisation data. The three scenarios tested in the need model were selected to illustrate the effects of no change in the age- and gender-specific need (resulting in the model predicting fewer required services and thus fewer GPs than observed) and the effects of increasing the need over time (more FTE GPs required than observed) and to match predicted and observed GP numbers (increasing utilisation rates). This highlights the value of ongoing collection of population need data and information on the appropriateness of the estimated utilisation rates. Without ‘gold standards’ for services required for different population groups with different levels of health need, the service norms used to determine the average level of services at baseline can affect the model outcomes. This then becomes an argument on what level of service is appropriate or optimal.

The best estimate of health need may be that determined under the second scenario, which represents a scenario in which utilisation rates have increased resulting in a reasonable balance of supply and demand. This is supported in some way by the reported low levels of unmet need found in Australia. The Patient Experience Survey reported that only 1.0% of people reported needing to see a GP but were not able in 2014–2015 and was similar in the previous 3 years with 1.2% in 2013–2014, 0.7% in 2012–2013 and 1.0% in 2011–2012 [44].

What the model does not accommodate is variation in the use of GP services. We know that the use of GP services is not uniform across Australia with higher rates of GP utilisation in some areas and among certain population groups and low rates in other areas such as rural and remote areas. Further research is required to address this limitation, but the model provides a basis for testing the effects of potential unmet need or over the use of services. Scenario analyses can test the effects of assuming under- or overuse within the sub-groups and corresponding policy initiatives to support increased or decreased service demand.

The scenario analysis for the need sub-model also illustrates the impact of changes in illness within the population on GP requirements. However, the challenge for the need-based models is to reliably predict changes in the morbidity of the population [45]. This model used the 2003 burden of disease study to estimate changes in incidence and prevalence cases over the projected period, and without more recent data, assumptions were made on how this may change (either based on demographic changes only or a small annual increase). An updated burden of disease study will be published in 2016, which may identify time-varying disease rates by age and gender over time. The model can be easily updated to reflect such data.

As with any simulation model, the model was not able to reflect the absolute reality of the supply of, and demand for, GP services over the modelled time horizon. Limitations relate to the quality or lack of data for both the supply and need sub-models. The population of the supply sub-model required longitudinal data describing the career paths of medical practitioners. At the time of the study, there is only one relevant dataset available in Australia—the MABEL longitudinal survey [30]. However, relying on these data has limitations in terms of sample size (especially when analysed by age, sex, work status and location) and representativeness (selection bias). The need for reliable and complete longitudinal data may be addressed in the future through the National Health Workforce Dataset (NHWD) and the Australian Health Practitioner Agency. Other supply-side data limitations included the following: the lack of consistency in definitions used for variables within and between datasets (e.g. use of general practitioner versus primary care practitioner), the lack of a unique qualification that captures the entire GP workforce and the inability to identify accurately sub-groups within a discipline such as overseas-trained doctors. This latter issue is particularly important in Australia, where immigrant doctors form an important component of the rural and remote GP workforce.

The need sub-model used prevalence and incidence data to determine the morbidity of the population, and this has a number of limitations. Firstly, these data relate only to illness in the community and do not capture other activities which require access to GP services. The model attempted to incorporate these other activities by including preventative and administrative tasks, but it is likely to have underestimated this component of GP care. Further research is needed to more accurately estimate this aspect of a GP’s workload. Secondly, the accuracy of the burden of disease data used to estimate incidence and prevalence will vary across categories due to the wide range of underlying data sources used. Also, these data do not capture all conditions managed by GPs. For example, ‘social problems’, which is an ICPC-2 chapter heading, consists of items such as relationship and housing problems for which there are no prevalence or incidence data, and so, such items were not included in the level of service estimates. While these make up a small proportion of GP activity (0.5% of problems managed in 2011–2012 [46]), their exclusion may underestimate the number of GP consultations required.

While the need sub-model accounted for differences in the number and duration of consultations, by age and sex, it was not possible to determine different consultation lengths for different conditions. The model assumed that consultations are of equal length across health conditions. However, it is known that consultation length does vary by condition. The BEACH research has reported that longer consultations are associated with problems in the psychological, social and female genital chapters. Medium-level consultations (>20 min) are associated with conditions in the neurological and endocrine/metabolic chapters, while problems in the respiratory, skin, eye and ear chapters were associated with shorter consultations [47, 48]. Similarly, the model did not account for the population having multiple conditions and the impact this may have on the level of service requirements. Some of the limitations could be addressed by further refinement of the model, for example, the model could incorporate disease-specific average consultation times. Other developments could include prediction of health needs by location to match predictions of supply by urban and rural location.

Despite these limitations, our results suggest that the model is credible and will usefully inform future GP workforce requirements. The next step will be to apply the model to forecasting GP requirements for SA over the next 20 years and assessing the effects of various policy and workforce scenarios, including changes in models of care.

## Conclusions

The workforce model developed by this study is the first to take incorporating health needs to estimating the future GP workforce in Australia. As noted by Health Workforce 2025, most models measure demand through service utilisation [8]. This study provides a methodology for determining demand based on the needs of the population, and it has done this for one professional group at a regional level. Despite numerous limitations with the datasets used, it has been possible to make use of prevalence and incidence data to estimate future health needs at a population level. Moreover, the model has used data collected through the BEACH Program to link incidence and prevalence to level of service. The scenario analysis provided alternative methods for determining the estimated level of service and GPs required to meet this service level and highlighted the effect of different assumptions on the model outcomes. This model can now be further refined to address some of the limitations in the methods and updated as better quality data become available (e.g. using the awaited update of the burden of disease in Australia). The model can also be used by policymakers and planners to assess the impact of scenarios such as changes in training places, demand for services and role substitution on the required size of the future GP workforce.

## Additional file

Additional file 1: Transition probabilities used in base year of the supply sub-model. The file contains the transition probabilities used in the base year of the supply sub-model for male and female GPs. (DOCX 41 kb)
