# Supplementary material for: Improving the planning of the GP workforce in Australia: a simulation model incorporating work transitions, health need and service usage
Source: Hum Resour Health. 2016 Apr 11;14:13. doi: 10.1186/s12960-016-0110-2 (PMC4828877; doi:10.1186/s12960-016-0110-2)
Supplement: Additional file 1: — Transition probabilities used in base year of the supply sub-model. The file contains the transition probabilities used in the base year of the supply sub-model for male and female GPs. (DOCX 41 kb) [file 12960_2016_110_MOESM1_ESM.docx]

### Additional file 1 – Transition probabilities used in base year of the supply sub-model

| **TRANSITION PROBABILITIES -MALES** | |  |
| --- | --- | --- |
| **From** | **To** | **2003-04** |
| <35 FT (urban) | FT (urban) | 0.928 |
| <35 FT (urban) | PT (urban) | 0.040 |
| <35 FT (urban) | FT (rural) | 0.032 |
| <35 FT (urban) | PT (rural) | 0.000 |
| <35 FT (urban) | Left workforce temp | 0.000 |
| <35 FT (urban) | Left workforce perm | 0.000 |
|  |  |  |
| <35 PT (urban) | PT (urban) | 0.888 |
| <35 PT (urban) | FT (urban) | 0.111 |
| <35 PT (urban) | PT (rural) | 0.000 |
| <35 PT (urban) | FT (rural) | 0.000 |
| <35 PT (urban) | Left workforce temp | 0.000 |
| <35 PT (urban) | Left workforce perm | 0.000 |
|  |  |  |
| 35-44 FT (urban) | FT (urban) | 0.934 |
| 35-44 FT (urban) | PT (urban) | 0.030 |
| 35-44 FT (urban) | FT (rural) | 0.036 |
| 35-44 FT (urban) | PT (rural) | 0.000 |
| 35-44 FT (urban) | Left workforce temp | 0.000 |
| 35-44 FT (urban) | Left workforce perm | 0.000 |
|  |  |  |
| 35-44 PT (urban) | PT (urban) | 0.793 |
| 35-44 PT (urban) | FT (urban) | 0.184 |
| 35-44 PT (urban) | PT (rural) | 0.013 |
| 35-44 PT (urban) | FT (rural) | 0.009 |
| 35-44 PT (urban) | Left workforce temp | 0.000 |
| 35-44 PT (urban) | Left workforce perm | 0.000 |
|  |  |  |
| 45-54 FT (urban) | FT (urban) | 0.960 |
| 45-54 FT (urban) | PT (urban) | 0.017 |
| 45-54 FT (urban) | FT (rural) | 0.022 |
| 45-54 FT (urban) | PT (rural) | 0.000 |
| 45-54 FT (urban) | Left workforce temp | 0.000 |
| 45-54 FT (urban) | Left workforce perm | 0.000 |
|  |  |  |
| 45-54 PT (urban) | PT (urban) | 0.81 |
| 45-54 PT (urban) | FT (urban) | 0.15 |
| 45-54 PT (urban) | PT (rural) | 0.02 |
| 45-54 PT (urban) | FT (rural) | 0.02 |
| 45-54 PT (urban) | Left workforce temp | 0.00 |
| 45-54 PT (urban) | Left workforce perm | 0.00 |
| 45-54 PT (urban) | 45-54 left workforce temp | 0.00 |
|  |  |  |
| 55-64 FT (urban) | FT (urban) | 0.937 |
| 55-64 FT (urban) | PT (urban) | 0.043 |
| 55-64 FT (urban) | FT (rural) | 0.020 |
| 55-64 FT (urban) | PT (rural) | 0.000 |
| 55-64 FT (urban) | Left workforce temp | 0.000 |
| 55-64 FT (urban) | Left workforce perm | 0.000 |
|  |  |  |
| 55-64 PT (urban) | PT (urban) | 0.896 |
| 55-64 PT (urban) | FT (urban) | 0.102 |
| 55-64 PT (urban) | PT (rural) | 0.001 |
| 55-64 PT (urban) | FT (rural) | 0.000 |
| 55-64 PT (urban) | Left workforce temp | 0.000 |
| 55-64 PT (urban) | Left workforce perm | 0.000 |
|  |  |  |
| 65+ FT (urban) | FT (urban) | 0.706 |
| 65+ FT (urban) | PT (urban) | 0.132 |
| 65+ FT (urban) | FT (rural) | 0.028 |
| 65+ FT (urban) | PT (rural) | 0.012 |
| 65+ FT (urban) | Left workforce temp | 0.000 |
| 65+ FT (urban) | Left workforce perm | 0.122 |
|  |  |  |
| 65+ PT (urban) | PT (urban) | 0.861 |
| 65+ PT (urban) | FT (urban) | 0.000 |
| 65+ PT (urban) | PT (rural) | 0.013 |
| 65+ PT (urban) | FT (rural) | 0.000 |
| 65+ PT (urban) | Left workforce temp | 0.000 |
| 65+ PT (urban) | Left workforce perm | 0.126 |
|  |  |  |
| <35 FT (rural) | FT (rural) | 0.885 |
| <35 FT (rural) | PT (rural) | 0.005 |
| <35 FT (rural) | FT (urban) | 0.099 |
| <35 FT (rural) | PT (urban) | 0.011 |
| <35 FT (rural) | Left workforce temp | 0.000 |
| <35 FT (rural) | Left workforce perm | 0.000 |
|  |  |  |
| <35 PT (rural) | PT (rural) | 0.759 |
| <35 PT (rural) | FT (rural) | 0.037 |
| <35 PT (rural) | PT (urban) | 0.154 |
| <35 PT (rural) | FT (urban) | 0.049 |
| <35 PT (rural) | Left workforce temp | 0.000 |
| <35 PT (rural) | Left workforce perm | 0.000 |
|  |  |  |
| 35-44 FT (rural) | FT (rural) | 0.904 |
| 35-44 FT (rural) | PT (rural) | 0.007 |
| 35-44 FT (rural) | FT (urban) | 0.085 |
| 35-44 FT (rural) | PT (urban) | 0.004 |
| 35-44 FT (rural) | Left workforce temp | 0.000 |
| 35-44 FT (rural) | Left workforce perm | 0.000 |
|  |  |  |
| 35-44 PT (rural) | PT (rural) | 0.835 |
| 35-44 PT (rural) | FT (rural) | 0.029 |
| 35-44 PT (rural) | PT (urban) | 0.111 |
| 35-44 PT (rural) | FT (urban) | 0.025 |
| 35-44 PT (rural) | Left workforce temp | 0.000 |
| 35-44 PT (rural) | Left workforce perm | 0.000 |
|  |  |  |
| 45-54 FT (rural) | FT (rural) | 0.919 |
| 45-54 FT (rural) | PT (rural) | 0.002 |
| 45-54 FT (rural) | FT (urban) | 0.077 |
| 45-54 FT (rural) | PT (urban) | 0.002 |
| 45-54 FT (rural) | Left workforce temp | 0.000 |
| 45-54 FT (rural) | Left workforce perm | 0.000 |
|  |  |  |
| 45-54 PT (rural) | PT (rural) | 0.848 |
| 45-54 PT (rural) | FT (rural) | 0.060 |
| 45-54 PT (rural) | PT (urban) | 0.076 |
| 45-54 PT (rural) | FT (urban) | 0.017 |
| 45-54 PT (rural) | Left workforce temp | 0.000 |
| 45-54 PT (rural) | Left workforce perm | 0.000 |
|  |  |  |
| 55-64 FT (rural) | FT (rural) | 0.830 |
| 55-64 FT (rural) | PT (rural) | 0.028 |
| 55-64 FT (rural) | FT (urban) | 0.131 |
| 55-64 FT (rural) | PT (urban) | 0.010 |
| 55-64 FT (rural) | Left workforce temp | 0.000 |
| 55-64 FT (rural) | Left workforce perm | 0.000 |
|  |  |  |
| 55-64 PT (rural) | PT (rural) | 0.747 |
| 55-64 PT (rural) | FT (rural) | 0.078 |
| 55-64 PT (rural) | PT (urban) | 0.141 |
| 55-64 PT (rural) | FT (urban) | 0.035 |
| 55-64 PT (rural) | Left workforce temp | 0.000 |
| 55-64 PT (rural) | Left workforce perm | 0.000 |
|  |  |  |
| 65+ FT (rural) | FT (rural) | 0.669 |
| 65+ FT (rural) | PT (rural) | 0.113 |
| 65+ FT (rural) | FT (urban) | 0.047 |
| 65+ FT (rural) | PT (urban) | 0.001 |
| 65+ FT (rural) | Left workforce temp | 0.037 |
| 65+ FT (rural) | Left workforce perm | 0.133 |
|  |  |  |
| 65+ PT (rural) | PT (rural) | 0.806 |
| 65+ PT (rural) | FT (rural) | 0.000 |
| 65+ PT (rural) | PT (urban) | 0.015 |
| 65+ PT (rural) | FT (urban) | 0.000 |
| 65+ PT (rural) | Left workforce temp | 0.040 |
| 65+ PT (rural) | Left workforce perm | 0.139 |
|  |  |  |
| **TEMPORARY EXITS** |  |  |
| <35 left workforce temp | FT (rural) | 0.000 |
| <35 left workforce temp | PT (rural) | 0.000 |
| <35 left workforce temp | FT (urban) | 0.000 |
| <35 left workforce temp | PT (urban) | 0.000 |
| <35 left workforce temp | Left workforce temp | 1.000 |
| <35 left workforce temp | Left workforce perm | 0.000 |
|  |  |  |
| 34-44 left workforce temp | FT (rural) | 0.000 |
| 34-44 left workforce temp | PT (rural) | 0.000 |
| 34-44 left workforce temp | FT (urban) | 0.000 |
| 34-44 left workforce temp | PT (urban) | 0.000 |
| 34-44 left workforce temp | Left workforce temp | 1.000 |
| 34-44 left workforce temp | Left workforce perm | 0.000 |
|  |  |  |
| 45-54 left workforce temp | FT (rural) | 0.000 |
| 45-54 left workforce temp | PT (rural) | 0.000 |
| 45-54 left workforce temp | FT (urban) | 0.000 |
| 45-54 left workforce temp | PT (urban) | 0.000 |
| 45-54 left workforce temp | Left workforce temp | 1.000 |
| 45-54 left workforce temp | Left workforce perm | 0.000 |
|  |  |  |
| 55-64 left workforce temp | FT (rural) | 0.000 |
| 55-64 left workforce temp | PT (rural) | 0.000 |
| 55-64 left workforce temp | FT (urban) | 0.000 |
| 55-64 left workforce temp | PT (urban) | 0.000 |
| 55-64 left workforce temp | Left workforce temp | 1.000 |
| 55-64 left workforce temp | Left workforce perm | 0.000 |
|  |  |  |
| 65+ left workforce temp | FT (rural) | 0.000 |
| 65+ left workforce temp | PT (rural) | 0.000 |
| 65+ left workforce temp | FT (urban) | 0.000 |
| 65+ left workforce temp | PT (urban) | 0.000 |
| 65+ left workforce temp | Left workforce temp | 1.000 |
| 65+ left workforce temp | Left workforce perm | 0.000 |
|  |  |  |
| **PERMANENT EXITS** |  |  |
| <35 permanently left workforce | permanently left workforce, move to next age group | 0.1 |
| <35 permanently left workforce | permanently left workforce, stay in same age group | 0.9 |
| 35-44 permanently left workforce | permanently left workforce, move to next age group | 0.1 |
| 35-44 permanently left workforce | permanently left workforce, stay in same age group | 0.9 |
| 45-54 permanently left workforce | permanently left workforce, move to next age group | 0.1 |
| 45-54 permanently left workforce | permanently left workforce, stay in same age group | 0.9 |
| 55-64 permanently left workforce | permanently left workforce, move to next age group | 0.1 |
| 55-64 permanently left workforce | permanently left workforce, stay in same age group | 0.9 |
| 65+ permanently left workforce | permanently left workforce, stay in same age group | 1 |

| **TRANSITION PROBABILITIES - FEMALES** | |  |
| --- | --- | --- |
| **From** | **To** | **2003-04** |
| <35 FT (urban) | FT (urban) | 0.932 |
| <35 FT (urban) | PT (urban) | 0.032 |
| <35 FT (urban) | FT (rural) | 0.036 |
| <35 FT (urban) | PT (rural) | 0.000 |
| <35 FT (urban) | Left workforce temp | 0.000 |
| <35 FT (urban) | Left workforce perm | 0.000 |
|  |  |  |
| <35 PT (urban) | PT (urban) | 0.890 |
| <35 PT (urban) | FT (urban) | 0.095 |
| <35 PT (urban) | PT (rural) | 0.008 |
| <35 PT (urban) | FT (rural) | 0.008 |
| <35 PT (urban) | Left workforce temp | 0.000 |
| <35 PT (urban) | Left workforce perm | 0.000 |
|  |  |  |
| 35-44 FT (urban) | FT (urban) | 0.768 |
| 35-44 FT (urban) | PT (urban) | 0.177 |
| 35-44 FT (urban) | FT (rural) | 0.031 |
| 35-44 FT (urban) | PT (rural) | 0.000 |
| 35-44 FT (urban) | Left workforce temp | 0.024 |
| 35-44 FT (urban) | Left workforce perm | 0.000 |
|  |  |  |
| 35-44 PT (urban) | PT (urban) | 0.966 |
| 35-44 PT (urban) | FT (urban) | 0.024 |
| 35-44 PT (urban) | PT (rural) | 0.009 |
| 35-44 PT (urban) | FT (rural) | 0.001 |
| 35-44 PT (urban) | Left workforce temp | 0.000 |
| 35-44 PT (urban) | Left workforce perm | 0.000 |
|  |  |  |
| 45-54 FT (urban) | FT (urban) | 0.957 |
| 45-54 FT (urban) | PT (urban) | 0.018 |
| 45-54 FT (urban) | FT (rural) | 0.017 |
| 45-54 FT (urban) | PT (rural) | 0.000 |
| 45-54 FT (urban) | Left workforce temp | 0.008 |
| 45-54 FT (urban) | Left workforce perm | 0.000 |
|  |  |  |
| 45-54 PT (urban) | PT (urban) | 0.952 |
| 45-54 PT (urban) | FT (urban) | 0.030 |
| 45-54 PT (urban) | PT (rural) | 0.004 |
| 45-54 PT (urban) | FT (rural) | 0.000 |
| 45-54 PT (urban) | Left workforce temp | 0.014 |
| 45-54 PT (urban) | Left workforce perm | 0.000 |
|  |  |  |
| 55-64 FT (urban) | FT (urban) | 0.642 |
| 55-64 FT (urban) | PT (urban) | 0.011 |
| 55-64 FT (urban) | FT (rural) | 0.019 |
| 55-64 FT (urban) | PT (rural) | 0.000 |
| 55-64 FT (urban) | Left workforce temp | 0.106 |
| 55-64 FT (urban) | Left workforce perm | 0.222 |
|  |  |  |
| 55-64 PT (urban) | PT (urban) | 0.840 |
| 55-64 PT (urban) | FT (urban) | 0.130 |
| 55-64 PT (urban) | PT (rural) | 0.014 |
| 55-64 PT (urban) | FT (rural) | 0.016 |
| 55-64 PT (urban) | Left workforce temp | 0.000 |
| 55-64 PT (urban) | Left workforce perm | 0.000 |
|  |  |  |
| 65+ FT (urban) | FT (urban) | 0.648 |
| 65+ FT (urban) | PT (urban) | 0.156 |
| 65+ FT (urban) | FT (rural) | 0.066 |
| 65+ FT (urban) | PT (rural) | 0.040 |
| 65+ FT (urban) | Left workforce temp | 0.000 |
| 65+ FT (urban) | Left workforce perm | 0.090 |
|  |  |  |
| 65+ PT (urban) | PT (urban) | 0.862 |
| 65+ PT (urban) | FT (urban) | 0.000 |
| 65+ PT (urban) | PT (rural) | 0.045 |
| 65+ PT (urban) | FT (rural) | 0.000 |
| 65+ PT (urban) | Left workforce temp | 0.000 |
| 65+ PT (urban) | Left workforce perm | 0.093 |
|  |  |  |
| <35 FT (rural) | FT (rural) | 0.925 |
| <35 FT (rural) | PT (rural) | 0.006 |
| <35 FT (rural) | FT (urban) | 0.068 |
| <35 FT (rural) | PT (urban) | 0.001 |
| <35 FT (rural) | Left workforce temp | 0.000 |
| <35 FT (rural) | Left workforce perm | 0.000 |
|  |  |  |
| <35 PT (rural) | PT (rural) | 0.753 |
| <35 PT (rural) | FT (rural) | 0.104 |
| <35 PT (rural) | PT (urban) | 0.104 |
| <35 PT (rural) | FT (urban) | 0.040 |
| <35 PT (rural) | Left workforce temp | 0.000 |
| <35 PT (rural) | Left workforce perm | 0.000 |
|  |  |  |
| 35-44 FT (rural) | FT (rural) | 0.889 |
| 35-44 FT (rural) | PT (rural) | 0.022 |
| 35-44 FT (rural) | FT (urban) | 0.084 |
| 35-44 FT (rural) | PT (urban) | 0.005 |
| 35-44 FT (rural) | Left workforce temp | 0.000 |
| 35-44 FT (rural) | Left workforce perm | 0.000 |
|  |  |  |
| 35-44 PT (rural) | PT (rural) | 0.673 |
| 35-44 PT (rural) | FT (rural) | 0.041 |
| 35-44 PT (rural) | PT (urban) | 0.089 |
| 35-44 PT (rural) | FT (urban) | 0.197 |
| 35-44 PT (rural) | Left workforce temp | 0.000 |
| 35-44 PT (rural) | Left workforce perm | 0.000 |
|  |  |  |
| 45-54 FT (rural) | FT (rural) | 0.888 |
| 45-54 FT (rural) | PT (rural) | 0.006 |
| 45-54 FT (rural) | FT (urban) | 0.100 |
| 45-54 FT (rural) | PT (urban) | 0.006 |
| 45-54 FT (rural) | Left workforce temp | 0.000 |
| 45-54 FT (rural) | Left workforce perm | 0.000 |
|  |  |  |
| 45-54 PT (rural) | PT (rural) | 0.631 |
| 45-54 PT (rural) | FT (rural) | 0.012 |
| 45-54 PT (rural) | PT (urban) | 0.207 |
| 45-54 PT (rural) | FT (urban) | 0.021 |
| 45-54 PT (rural) | Left workforce temp | 0.130 |
| 45-54 PT (rural) | Left workforce perm | 0.000 |
|  |  |  |
| 55-64 FT (rural) | FT (rural) | 0.596 |
| 55-64 FT (rural) | PT (rural) | 0.015 |
| 55-64 FT (rural) | FT (urban) | 0.073 |
| 55-64 FT (rural) | PT (urban) | 0.003 |
| 55-64 FT (rural) | Left workforce temp | 0.106 |
| 55-64 FT (rural) | Left workforce perm | 0.206 |
|  |  |  |
| 55-64 PT (rural) | PT (rural) | 0.814 |
| 55-64 PT (rural) | FT (rural) | 0.040 |
| 55-64 PT (rural) | PT (urban) | 0.111 |
| 55-64 PT (rural) | FT (urban) | 0.035 |
| 55-64 PT (rural) | Left workforce temp | 0.000 |
| 55-64 PT (rural) | Left workforce perm | 0.000 |
|  |  |  |
| 65+ FT (rural) | FT (rural) | 0.708 |
| 65+ FT (rural) | PT (rural) | 0.123 |
| 65+ FT (rural) | FT (urban) | 0.018 |
| 65+ FT (rural) | PT (urban) | 0.009 |
| 65+ FT (rural) | Left workforce temp | 0.037 |
| 65+ FT (rural) | Left workforce perm | 0.105 |
|  |  |  |
| 65+ PT (rural) | PT (rural) | 0.867 |
| 65+ PT (rural) | FT (rural) | 0.000 |
| 65+ PT (rural) | PT (urban) | 0.021 |
| 65+ PT (rural) | FT (urban) | 0.000 |
| 65+ PT (rural) | Left workforce temp | 0.032 |
| 65+ PT (rural) | Left workforce perm | 0.081 |
|  |  |  |
|  |  |  |
|  |  |  |
| **TEMPORARY EXITS** |  |  |
| <35 left workforce temp | FT (rural) | 0.000 |
| <35 left workforce temp | PT (rural) | 0.000 |
| <35 left workforce temp | FT (urban) | 0.000 |
| <35 left workforce temp | PT (urban) | 0.000 |
| <35 left workforce temp | Left workforce temp | 1.000 |
| <35 left workforce temp | Left workforce perm | 0.000 |
|  |  |  |
| 34-44 left workforce temp | FT (rural) | 0.000 |
| 34-44 left workforce temp | PT (rural) | 0.000 |
| 34-44 left workforce temp | FT (urban) | 0.000 |
| 34-44 left workforce temp | PT (urban) | 0.000 |
| 34-44 left workforce temp | Left workforce temp | 1.000 |
| 34-44 left workforce temp | Left workforce perm | 0.000 |
|  |  |  |
| 45-54 left workforce temp | FT (rural) | 0.000 |
| 45-54 left workforce temp | PT (rural) | 0.000 |
| 45-54 left workforce temp | FT (urban) | 0.000 |
| 45-54 left workforce temp | PT (urban) | 0.000 |
| 45-54 left workforce temp | Left workforce temp | 1.000 |
| 45-54 left workforce temp | Left workforce perm | 0.000 |
|  |  |  |
| 55-64 left workforce temp | FT (rural) | 0.000 |
| 55-64 left workforce temp | PT (rural) | 0.000 |
| 55-64 left workforce temp | FT (urban) | 0.000 |
| 55-64 left workforce temp | PT (urban) | 0.000 |
| 55-64 left workforce temp | Left workforce temp | 1.000 |
| 55-64 left workforce temp | Left workforce perm | 0.000 |
|  |  |  |
| 65+ left workforce temp | FT (rural) | 0.000 |
| 65+ left workforce temp | PT (rural) | 0.000 |
| 65+ left workforce temp | FT (urban) | 0.000 |
| 65+ left workforce temp | PT (urban) | 0.000 |
| 65+ left workforce temp | Left workforce temp | 1.000 |
| 65+ left workforce temp | Left workforce perm | 0.000 |
|  |  |  |
|  |  |  |
| **PERMANENT EXITS** |  |  |
| <35 permanently left workforce | permanently left workforce, move to next age group | 0.1 |
| <35 permanently left workforce | permanently left workforce, stay in same age group | 0.9 |
| 35-44 permanently left workforce | permanently left workforce, move to next age group | 0.1 |
| 35-44 permanently left workforce | permanently left workforce, stay in same age group | 0.9 |
| 45-54 permanently left workforce | permanently left workforce, move to next age group | 0.1 |
| 45-54 permanently left workforce | permanently left workforce, stay in same age group | 0.9 |
| 55-64 permanently left workforce | permanently left workforce, move to next age group | 0.1 |
| 55-64 permanently left workforce | permanently left workforce, stay in same age group | 0.9 |
| 65+ permanently left workforce | permanently left workforce, stay in same age group | 1 |
